# Supplementary material for: De Novo Prediction of Drug Targets and Candidates by Chemical Similarity-Guided Network-Based Inference
Source: Int J Mol Sci. 2022 Aug 26;23(17):9666. doi: 10.3390/ijms23179666 (PMC9455815; doi:10.3390/ijms23179666)
Supplement: Supplementary file 1 [file ijms-23-09666-s001.zip › ijms-1820355-supplementary.pdf]

De novo prediction of drug targets and candidates by chemical similarity-guided network-based inference: Supplementary information

Carlos Vigil-Vásquez and Andreas Schüller

S1 *de novo* network-based inference method

Denoting a set of $N_{\mathcal{F}}$ features as $F = \{f_1, f_2, ..., f_{N_{\mathcal{F}-1}}, f_{N_{\mathcal{F}}}\}$, a set of $N_{\mathcal{C}}$ novel compounds without known targets as $\mathcal{C} = \{c_1, c_2, ..., c_{N_{\mathcal{C}-1}}, c_{N_{\mathcal{C}}}\}$, a set of $N_{\mathcal{D}}$ drugs with known targets as $\mathcal{D} = \{d_1, d_2, ..., d_{N_{\mathcal{D}-1}}, d_{N_{\mathcal{D}}}\}$, and a set of $N\mathcal{T}$ biological targets as $\mathcal{T} = \{t_1, t_2, ..., t_{N_{\mathcal{T}-1}}, t_{N_{\mathcal{T}}}\}$, a compound-feature-drug-target graph can be represented as the graph $G(V, E)$, where $V = \mathcal{C} \cup \mathcal{F} \cup \mathcal{D} \cup \mathcal{T}$ is the node set and $E = E_{\mathcal{FC}} \cup E_{\mathcal{FD}} \cup E_{\mathcal{DT}}$ is the edge set constructed from the edges between features and novel compounds, between features and drugs and between drugs and targets, respectively.

The initial resources in graph G can be represented as a symmetric adjacency matrix of order $N_{\mathcal{C}} + N_{\mathcal{F}} + N_{\mathcal{D}} + N_{\mathcal{T}}$ given by

$$\mathbf{R} = \begin{bmatrix} 0 & 0 & \mathbf{M}_{\mathcal{CF}} & 0 \\ 0 & 0 & \mathbf{M}_{\mathcal{DF}} & \mathbf{M}_{\mathcal{DT}} \\ \mathbf{M}^{\intercal}_{\mathcal{CF}} & \mathbf{M}^{\intercal}_{\mathcal{DF}} & 0 & 0 \\ 0 & \mathbf{M}^{\intercal}_{\mathcal{DT}} & 0 & 0 \end{bmatrix} \tag{1}$$

where $\mathbf{M}_{\mathcal{CF}}$ is the adjacency matrix for the interactions between compounds and features, $\mathbf{M}_{\mathcal{DF}}$ is the adjacency matrix for the interactions between drugs and features and $\mathbf{M}_{\mathcal{DT}}$ is the adjacency matrix for the interactions between drugs and biological targets.

From $\mathbf{R}$, one can construct an adjacency matrix for a graph that only contains drugs with known interactions with targets and the features for the selected drugs. This adjacency matrix, that we will denote with $\mathbf{R_0}$, is given by

$$\mathbf{R_0} = \begin{bmatrix} 0 & 0 & 0 & 0 \\ 0 & 0 & \mathbf{M}_{\mathcal{DF}} & \mathbf{M}_{\mathcal{DT}} \\ 0 & \mathbf{M}^{\intercal}_{\mathcal{DF}} & 0 & 0 \\ 0 & \mathbf{M}^{\intercal}_{\mathcal{DT}} & 0 & 0 \end{bmatrix} \tag{2}$$

From $\mathbf{R_0}$, the transfer matrix $\mathbf{W}$ of order $N_{\mathcal{C}} + N_{\mathcal{F}} + N_{\mathcal{D}} + N_{\mathcal{T}}$ is defined by

$$\mathbf{W}(i,j) = \begin{cases} \frac{\mathbf{R_0}(i,j)}{\sum_{l=1}^{N_{\mathcal{F}}+N_{\mathcal{C}}+N_{\mathcal{D}}+N_{\mathcal{T}}} \mathbf{R_0}(i,l)}, & \text{if } \sum_{l=1}^{N_{\mathcal{F}}+N_{\mathcal{C}}+N_{\mathcal{D}}+N_{\mathcal{T}}} \mathbf{R_0}(i,l) \neq 0, \\ 0, & \text{otherwise.} \end{cases} \tag{3}$$

The final resource matrix $\mathbf{R_1}$, which correspond to the predicted targets for all the chemical compounds present in the graph G is obtained by

$$\mathbf{R_1} = \mathbf{R} \times \mathbf{W}^k \tag{4}$$

where $\mathbf{R}$ is the initial resource matrix, $\mathbf{W}$ is the transfer matrix and k is the number of resource spreading processes. Based on previous studies, the number of resource-spreading processes used in this study is equal to 2 ($k = 2$).

S2 Dataset preparation

S2.1 ChEMBL datasets entries selection criteria

From ChEMBL versions 24 (June, 2018) and 28 (February, 2021), all interactions that meet the following criteria are collected:

1. Species identifier equals Homo Sapiens;
2. Assay type equals ”protein-ligand binding”;
3. K_i, K_d, IC_{50} or EC_{50} between 0 μM and less than 10 μM;
4. Molecule must have more than 5 and less than 80 heavy atoms;
5. Activity comment must denote that the ligand is active for annotated target;
6. Activity value must be ”minor”, ”much less”, ”less than or equal to”, ”equal” or ”equal to equal” (represented as ”<”, ”<<”, ”<=”, ”=”, and ”==”, respectively) to the value obtained experimentally and
7. Ligand must have a maximum clinical phase equal to or greater than 1.

S2.2 Tanimoto distributions

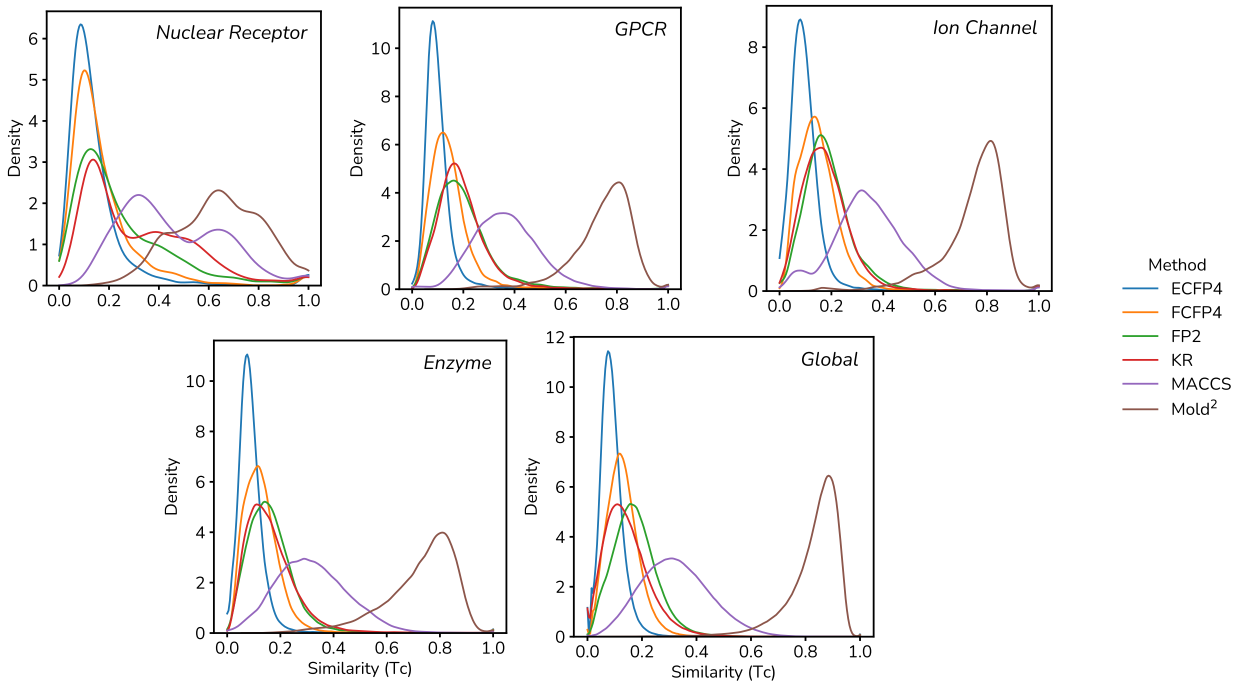

Figure S1: **Tanimoto distribution of the studied datasets and fingerprints.** Kernel density estimate (KDE) of the chemical similarity observed for each molecular descriptor throughout the 5 internal validation datasets

S2.3 Additional information for molecular descriptors

Mold^2 descriptor was obtained online from `https://www.fda.gov/science-research/bioinformatics-tools/mold2`, accessed on 20 March 2022.

S3 Evaluation metrics description

S3.1 Overall performance

S3.1.1 Area under the Receiver Operating Characteristic curve (AuROC)

To calculate the area under the receiver operating characteristic curve (AuROC), first we calculate the true-positive rate (TPR) and false-positive rate (FPR) for each discrimination thresholds to then calculate the area under the curve constructed from this values. TPR and FPR are given by

$$TPR = \frac{TP}{TP + FN} \tag{5}$$

$$FPR = \frac{FP}{FP + TN} \tag{6}$$

where TP corresponds to the true-positive predictions, TN to the true-negative predictions, FP to the false-positive predictions and FN to the false-negative predictions.

This metric was calculated using the function `roc_auc_score` from the scikit-learn Python package.

S3.1.2 Area under the Precision-Recall curve (AuPRC)

To calculate the area under the precision-recall curve (AuPRC), first we calculate the precision and recall for each discrimination thresholds to then calculate the area under the curve constructed from this values. Precision and recall are given by

$$\text{Precision} = \frac{TP}{TP + FP} \tag{7}$$

$$\text{Recall} = \frac{TP}{TP + FN} \tag{8}$$

where TP corresponds to the true-positive predictions, FP to the false-positive predictions and FN to the false-negative predictions.

This metric was calculated using the function `average_precision_score` from the scikit-learn Python package.

S3.2 Early-recognition performance

In virtual screening, only the best predictions obtained from a model are selected for posterior experimental validation. Therefore, understanding the predictive performance of a model for these predictions is essential to (1) make accurate predictions that will translate to biological activity and (2) understand the limitations of the model. The metrics discussed here can be evaluated at a given cut-off rank, considering only the topmost results returned by the predictive method, hence informing of the predictive performance of the model for only the best predictions.

S3.2.1 Precision and Recall at L (P@L and R@L)

These metrics correspond to the precision and recall of the best L predictions obtained from a model. In this study we calculate these metrics over the ligands, i.e., we calculate the precision and recall for the best L prediction for each ligand in the test set and average them to have a single score. Precision at L (P@L) and Recall at L (R@L) are given by

$$P@L = \frac{1}{N_D} \sum_{i=1}^{D} \frac{X_i(L)}{L} \tag{9}$$

$$R@L = \frac{1}{N_D} \sum_{i=1}^{D} \frac{X_i(L)}{X_i} \tag{10}$$

where L if the number of top prediction to evaluate, ND corresponds to the number of ligands that participated in the evaluation (equal to 1 in leave-one-out cross-validation), Xi is the number of drug-target interactions removed for a given drug in the data splitting procedure and Xi(L) are the number of true-positives in the top L predictions. In this study we employed a length of the list equal to 10 (L = 20) based in previous studies [1–5].

This metric was calculated using a in-house implementation in Python.

S3.2.2 Boltzmann-Enhanced Discrimination of ROC (AuBEDROC)

BEDROC corresponds to a transformation of the commonly used Receiver Operating Characteristic in order to quantify the early recognition capability of a predictive model. This metric differentially weights the entries based on their position in the predictions list, giving more importance to the first entries in a list, i.e., the ones with higher probabilities or predictive scores. BEDROC is given by

$$BEDROC = \frac{RIE}{\alpha} + \frac{1}{1 - e^{\alpha}} \tag{11}$$

where RIE corresponds to the robust initial enhancement proposed by Sheridan et al. (2001) and corresponds to the early recognition weighting parameter. Based in previous studies [6], a value of 20 was employed for α.

This metric was calculated using a in-house implementation in Python.

S3.3 Binary prediction performance

The last common practice in virtual screening is to assign a score or probability threshold for the predictions obtained from a model and manually select or cherry-pick predictions for experimental validation. In order to evaluate the predictive performance under this paradigm, we calculated the score for each metric at all possible discrimination thresholds and then retrieved the maximum observed value.

S3.3.1 Matthews correlation coefficient (MCC)

Evaluation metric that measures the correlation between the true and predicted binary classification, where values of -1 indicate total disagreement between truth and prediction, 0 indicates a random prediction and +1 indicates total agreement between truth and prediction. MCC is given by

$$MCC = \frac{TP \times TN - FP \times FN}{\sqrt{(TP + FP)(TP + FN)(TN + FP)(TN + FN)}} \tag{12}$$

where TP corresponds to the true-positive predictions, TN to the true-negative predictions, FP to the false-positive predictions and FN to the false-negative predictions.

This metric was calculated using a in-house implementation in Python.

S3.3.2 F$_1$ score (F_1)

Evaluation metric that measures the classification performance as the harmonic mean between the precision and recall scores. The highest possible value of an F-score is 1.0, indicating perfect precision and recall, and the lowest possible value is 0, if either the precision or the recall is zero. F1 score is given by

$$F_1 = \frac{TP}{TP + \frac{1}{2}(FP + FN)} \tag{13}$$

where TP corresponds to the true-positive predictions, FP to the false-positive predictions and FN to the false-negative predictions.

This metric was calculated using a in-house implementation in Python.

S3.3.3 Balanced Accuracy (bACC)

Balanced accuracy (bACC) corresponds to the arithmetic mean of sensitivity (also known as true-positive rate or recall) and specificity (also known as true-negative rate) and its use case is assessing binary or multi-class classification performance in imbalanced datasets, i.e., one of the classes is over-represented in the dataset. bACC is given by

$$bACC = \frac{TPR + TNR}{2} \tag{14}$$

where TPR corresponds to the true-positive rate and TNR to the true-negative rate.

True-negative rate (TNR) is given by

$$TNR = \frac{TN}{TN + FP} \tag{15}$$

where TN corresponds to the true-negative predictions and FP to the false-positive predictions.

This metric was calculated using a in-house implementation in Python.

S4 Predictive performance

S4.1 Leave-one-out cross-validation

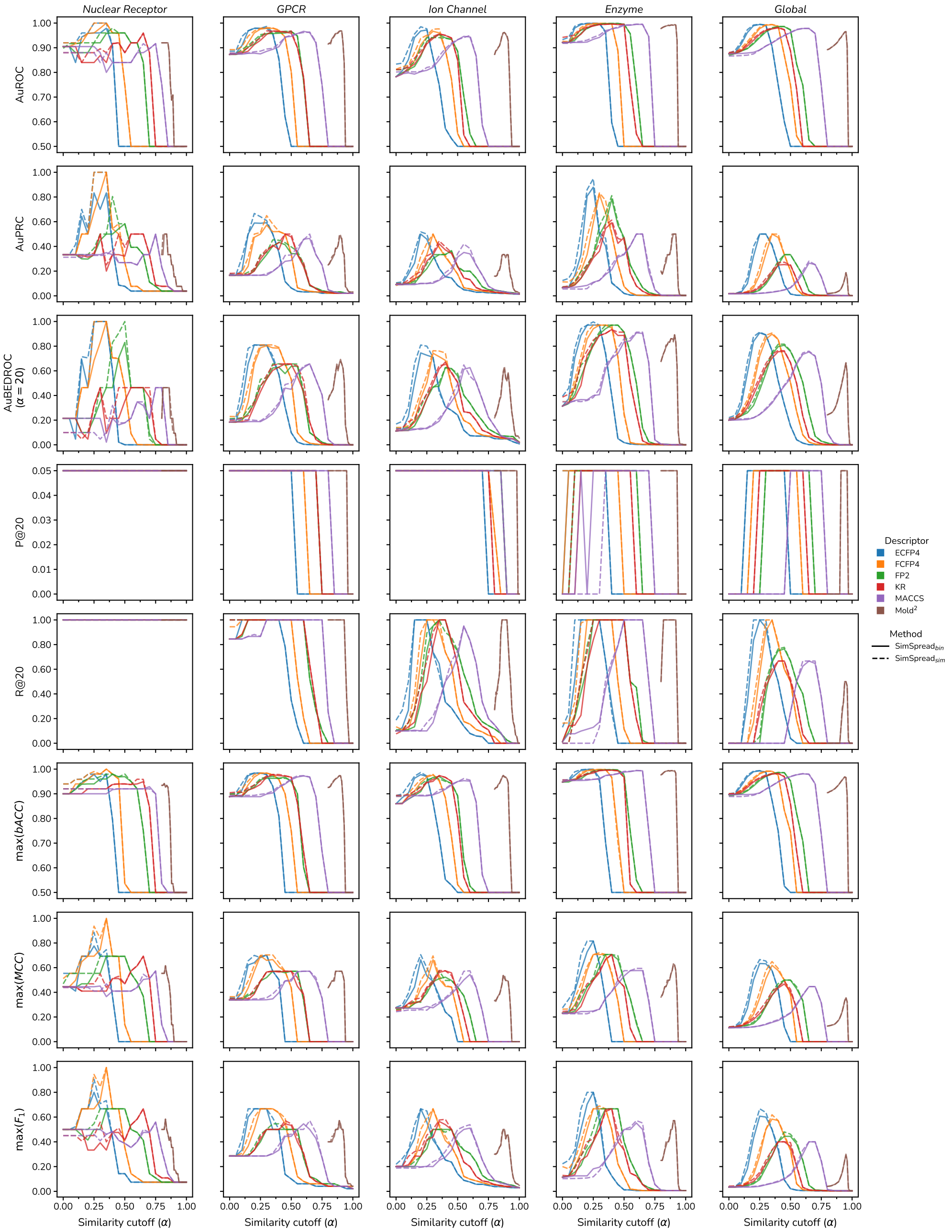

Figure S2: α **parameter optimization over LOO CV.** Median score for metrics obtained for LOO CV over 5 datasets using values between 0 and 1 with a step size of 0.05. A total of six different molecular descriptors were used for the method optimization: ECFP4 (blue), FCFP4 (orange), FP2 (green), KR (red), MACCS keys (purple) and Mold2 (brown). Solid lines correspond to SimSpread$_{bin}$ while dashed lines correspond to SimSpread$_{sim}$.

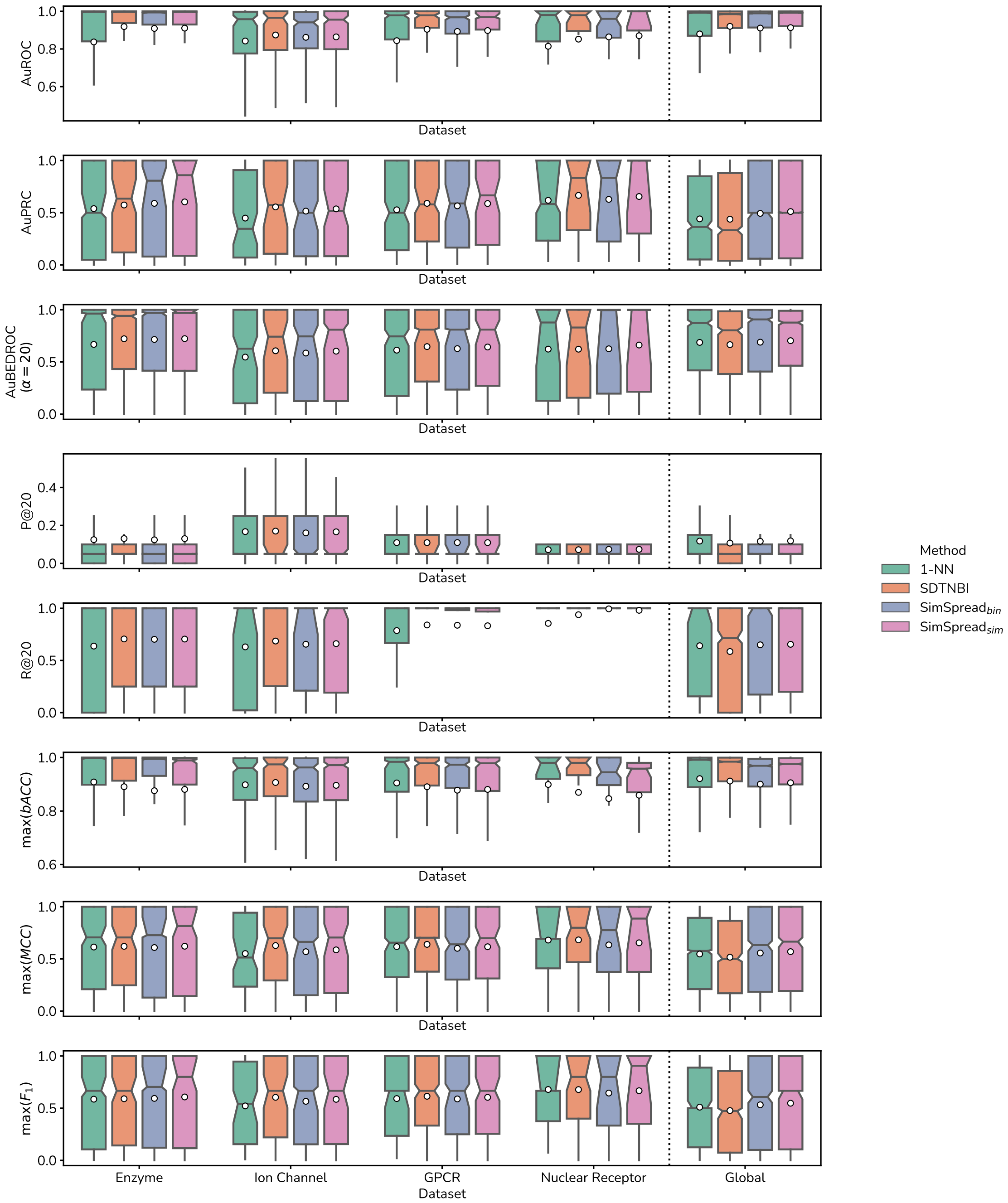

Figure S3: **Predictive performance comparison between the studied methods in LOO CV.** Boxplots for AuROC, AuPRC, AuBEDROC, P@20, R@20, max(MCC), max(F_1) and max(bACC) obtained in the LOO CV over the five validation datasets for 1-NN, NBI, SDTNBI, SimSpread_{bin} and SimSpread_{sim} using ECFP4 molecular descriptor and Tanimoto coefficient as similarity measure. For SimSpread variants, the similarity cutoff is equal to the optimal cutoff that maximizes the performance of the method for that metric. Boxplot notch represents the bootstrapped 95% CI around the median, whiskers correspond to 1.5x IQR and white dot represents the mean.

Table S1: **Overall average performance for the studied methods in LOO CV.** Mean ± SD for AuROC and AuPRC observed in LOO CV over the five validation datasets using the ECFP4 molecular descriptor and Tanimoto coefficient as chemical similarity measure. For SimSpread variants, the similarity cutoff is equal to the optimal cutoff that maximizes the mean performance of the method for that metric.

Dataset	Method	AuROC	AuPRC
NR	NN	0.814 ± 0.326	0.619 ± 0.377
	SDTNBI	0.852 ± 0.284	0.667 ± 0.373
	SimSpread_{bin}	0.864 ± 0.209	0.628 ± 0.401
	SimSpread_{sim}	0.870 ± 0.210	0.656 ± 0.397
IC	NN	0.842 ± 0.231	0.449 ± 0.392
	SDTNBI	0.874 ± 0.186	0.556 ± 0.409
	SimSpread_{bin}	0.862 ± 0.183	0.516 ± 0.411
	SimSpread_{sim}	0.864 ± 0.192	0.537 ± 0.416
GPCR	NN	0.844 ± 0.277	0.528 ± 0.385
	SDTNBI	0.905 ± 0.177	0.590 ± 0.376
	SimSpread_{bin}	0.893 ± 0.175	0.566 ± 0.397
	SimSpread_{sim}	0.898 ± 0.174	0.588 ± 0.400
Enzyme	NN	0.837 ± 0.299	0.540 ± 0.435
	SDTNBI	0.919 ± 0.168	0.574 ± 0.417
	SimSpread_{bin}	0.909 ± 0.184	0.590 ± 0.428
	SimSpread_{sim}	0.911 ± 0.184	0.604 ± 0.428
Global	NN	0.880 ± 0.231	0.442 ± 0.385
	SDTNBI	0.920 ± 0.151	0.438 ± 0.396
	SimSpread_{bin}	0.911 ± 0.168	0.494 ± 0.400
	SimSpread_{sim}	0.913 ± 0.168	0.512 ± 0.405

Table S2: **Overall median performance for the studied methods in LOO CV.** Q1, median and Q3 for AuROC and AuPRC observed in LOO CV over the five validation datasets using the ECFP4 molecular descriptor and Tanimoto coefficient as chemical similarity measure. For SimSpread variants, the similarity cutoff is equal to the optimal cutoff that maximizes the mean performance of the method for that metric.

		AuROC			AuPRC		
Dataset	Method	Q1	median	Q3	Q1	median	Q3
NR	NN	0.840	0.980	1.000	0.233	0.583	1.000
	SDTNBI	0.895	0.979	1.000	0.333	0.833	1.000
	SimSpread$_{bin}$	0.860	0.960	1.000	0.225	0.833	1.000
	SimSpread$_{sim}$	0.898	1.000	1.000	0.301	1.000	1.000
IC	NN	0.776	0.958	0.998	0.071	0.347	0.909
	SDTNBI	0.795	0.966	1.000	0.108	0.574	1.000
	SimSpread$_{bin}$	0.803	0.941	0.996	0.083	0.500	1.000
	SimSpread$_{sim}$	0.798	0.956	1.000	0.084	0.518	1.000
GPCR	NN	0.850	0.978	1.000	0.142	0.500	1.000
	SDTNBI	0.913	0.979	1.000	0.225	0.580	1.000
	SimSpread$_{bin}$	0.882	0.968	1.000	0.167	0.589	1.000
	SimSpread$_{sim}$	0.903	0.969	1.000	0.193	0.667	1.000
Enzyme	NN	0.840	0.997	1.000	0.050	0.500	1.000
	SDTNBI	0.938	0.997	1.000	0.120	0.635	1.000
	SimSpread$_{bin}$	0.929	0.994	1.000	0.081	0.807	1.000
	SimSpread$_{sim}$	0.930	0.997	1.000	0.088	0.860	1.000
Global	NN	0.870	0.992	1.000	0.053	0.364	0.850
	SDTNBI	0.911	0.985	0.999	0.040	0.333	0.880
	SimSpread$_{bin}$	0.914	0.989	0.999	0.060	0.500	1.000
	SimSpread$_{sim}$	0.921	0.992	1.000	0.063	0.500	1.000

Table S3: **Early-recognition average performance for the studied methods in LOO CV.** Mean ± SD for AuBEDROC, P@20 and R@20 observed in LOO CV over the five validation datasets using the ECFP4 molecular descriptor and Tanimoto coefficient as chemical similarity measure. For SimSpread variants, the similarity cutoff is equal to the optimal cutoff that maximizes the performance of the method for that metric.

Dataset	Method	AuBEDROC	P@20	R@20
NR	NN	0.623 ± 0.423	0.072 ± 0.073	0.855 ± 0.344
	SDTNBI	0.622 ± 0.418	0.072 ± 0.069	0.938 ± 0.238
	SimSpread_{bin}	0.626 ± 0.425	0.074 ± 0.063	0.994 ± 0.028
	SimSpread_{sim}	0.662 ± 0.414	0.074 ± 0.072	0.980 ± 0.098
IC	NN	0.547 ± 0.406	0.167 ± 0.222	0.630 ± 0.439
	SDTNBI	0.607 ± 0.398	0.170 ± 0.199	0.686 ± 0.408
	SimSpread_{bin}	0.585 ± 0.408	0.161 ± 0.211	0.655 ± 0.415
	SimSpread_{sim}	0.604 ± 0.411	0.166 ± 0.217	0.661 ± 0.424
GPCR	NN	0.613 ± 0.393	0.109 ± 0.127	0.786 ± 0.372
	SDTNBI	0.647 ± 0.371	0.109 ± 0.122	0.840 ± 0.328
	SimSpread_{bin}	0.628 ± 0.391	0.110 ± 0.125	0.837 ± 0.331
	SimSpread_{sim}	0.643 ± 0.391	0.109 ± 0.127	0.832 ± 0.336
Enzyme	NN	0.668 ± 0.408	0.125 ± 0.223	0.638 ± 0.455
	SDTNBI	0.722 ± 0.369	0.131 ± 0.218	0.706 ± 0.423
	SimSpread_{bin}	0.716 ± 0.390	0.124 ± 0.215	0.703 ± 0.426
	SimSpread_{sim}	0.723 ± 0.392	0.131 ± 0.225	0.705 ± 0.425
Global	NN	0.687 ± 0.362	0.118 ± 0.166	0.641 ± 0.421
	SDTNBI	0.666 ± 0.348	0.107 ± 0.166	0.586 ± 0.432
	SimSpread_{bin}	0.690 ± 0.374	0.116 ± 0.169	0.650 ± 0.421
	SimSpread_{sim}	0.704 ± 0.343	0.119 ± 0.174	0.655 ± 0.419

Table S4: **Early-recognition median performance for the studied methods in LOO CV.** Q1, median and Q3 for AuBEDROC, P@20 and R@20 observed in LOO CV over the five validation datasets using the ECFP4 molecular descriptor and Tanimoto coefficient as chemical similarity measure. For SimSpread variants, the similarity cutoff is equal to the optimal cutoff that maximizes the performance of the method for that metric.

		AuBEDROC			P@20			R@20		
Dataset	Method	Q1	median	Q3	Q1	median	Q3	Q1	median	Q3
NR	NN	0.128	0.878	1.000	0.05	0.05	0.10	1.000	1.000	1.000
	SDTNBI	0.157	0.830	1.000	0.05	0.05	0.10	1.000	1.000	1.000
	SimSpread_{bin}	0.196	0.996	1.000	0.05	0.05	0.10	1.000	1.000	1.000
	SimSpread_{sim}	0.215	1.000	1.000	0.05	0.05	0.10	1.000	1.000	1.000
IC	NN	0.103	0.627	1.000	0.05	0.05	0.25	0.022	1.000	1.000
	SDTNBI	0.205	0.742	1.000	0.05	0.05	0.25	0.254	1.000	1.000
	SimSpread_{bin}	0.125	0.745	1.000	0.05	0.05	0.25	0.211	1.000	1.000
	SimSpread_{sim}	0.126	0.809	1.000	0.05	0.05	0.25	0.192	1.000	1.000
GPCR	NN	0.174	0.745	1.000	0.05	0.05	0.15	0.667	1.000	1.000
	SDTNBI	0.312	0.810	1.000	0.05	0.05	0.15	1.000	1.000	1.000
	SimSpread_{bin}	0.235	0.810	1.000	0.05	0.05	0.15	0.982	1.000	1.000
	SimSpread_{sim}	0.272	0.810	1.000	0.05	0.05	0.15	0.969	1.000	1.000
Enzyme	NN	0.236	0.964	1.000	0.00	0.05	0.10	0.000	1.000	1.000
	SDTNBI	0.433	0.942	1.000	0.05	0.05	0.10	0.250	1.000	1.000
	SimSpread_{bin}	0.417	0.970	1.000	0.00	0.05	0.10	0.250	1.000	1.000
	SimSpread_{sim}	0.416	0.970	1.000	0.00	0.05	0.10	0.250	1.000	1.000
Global	NN	0.419	0.873	1.000	0.05	0.05	0.15	0.156	1.000	1.000
	SDTNBI	0.385	0.803	0.986	0.00	0.05	0.10	0.000	0.714	1.000
	SimSpread_{bin}	0.408	0.908	1.000	0.05	0.05	0.10	0.173	1.000	1.000
	SimSpread_{sim}	0.464	0.877	0.990	0.05	0.05	0.10	0.200	1.000	1.000

Table S5: **Binary prediction average performance for the studied methods in LOO CV.** Mean ± SD for max(bACC), max(MCC) and max(F_1) observed in LOO CV over the five validation datasets using the ECFP4 molecular descriptor and Tanimoto coefficient as chemical similarity measure. For SimSpread variants, the similarity cutoff is equal to the optimal cutoff that maximizes the performance of the method for that metric.

Dataset	Method	max(bACC)	max(MCC)	max(F_1)
NR	NN	0.899 ± 0.164	0.681 ± 0.348	0.679 ± 0.342
	SDTNBI	0.870 ± 0.255	0.683 ± 0.361	0.678 ± 0.362
	SimSpread_{bin}	0.847 ± 0.262	0.635 ± 0.410	0.645 ± 0.387
	SimSpread_{sim}	0.859 ± 0.244	0.655 ± 0.413	0.667 ± 0.387
IC	NN	0.898 ± 0.137	0.552 ± 0.349	0.522 ± 0.375
	SDTNBI	0.907 ± 0.146	0.628 ± 0.355	0.604 ± 0.381
	SimSpread_{bin}	0.893 ± 0.157	0.569 ± 0.397	0.566 ± 0.392
	SimSpread_{sim}	0.896 ± 0.160	0.587 ± 0.403	0.585 ± 0.398
GPCR	NN	0.905 ± 0.145	0.618 ± 0.341	0.592 ± 0.359
	SDTNBI	0.891 ± 0.225	0.640 ± 0.336	0.614 ± 0.358
	SimSpread_{bin}	0.878 ± 0.233	0.602 ± 0.380	0.589 ± 0.382
	SimSpread_{sim}	0.881 ± 0.233	0.617 ± 0.383	0.604 ± 0.386
Enzyme	NN	0.909 ± 0.159	0.614 ± 0.394	0.586 ± 0.416
	SDTNBI	0.891 ± 0.247	0.621 ± 0.382	0.590 ± 0.405
	SimSpread_{bin}	0.876 ± 0.260	0.609 ± 0.417	0.594 ± 0.423
	SimSpread_{sim}	0.881 ± 0.248	0.622 ± 0.418	0.608 ± 0.424
Global	NN	0.921 ± 0.128	0.547 ± 0.351	0.510 ± 0.374
	SDTNBI	0.912 ± 0.186	0.516 ± 0.357	0.477 ± 0.382
	SimSpread_{bin}	0.900 ± 0.187	0.557 ± 0.377	0.533 ± 0.388
	SimSpread_{sim}	0.906 ± 0.187	0.570 ± 0.381	0.548 ± 0.392

Table S6: **Binary prediction median performance for the studied methods in LOO CV.** Q1, median and Q3 for max(bACC), max(MCC) and max(F_1) observed in LOO CV over the five validation datasets using the ECFP4 molecular descriptor and Tanimoto coefficient as chemical similarity measure. For SimSpread variants, the similarity cutoff is equal to the optimal cutoff that maximizes the performance of the method for that metric.

		max(bACC)			max(MCC)			max(F_1)		
Dataset	Method	Q1	median	Q3	Q1	median	Q3	Q1	median	Q3
NR	NN	0.920	0.980	1.000	0.410	0.693	1.000	0.375	0.667	1.000
	SDTNBI	0.934	0.980	1.000	0.469	0.799	1.000	0.400	0.800	1.000
	SimSpread_{bin}	0.897	0.945	1.000	0.376	0.776	1.000	0.333	0.800	1.000
	SimSpread_{sim}	0.870	0.958	0.980	0.376	0.887	1.000	0.350	0.906	1.000
IC	NN	0.842	0.961	0.998	0.235	0.514	0.943	0.155	0.542	0.947
	SDTNBI	0.855	0.974	1.000	0.294	0.698	1.000	0.220	0.667	1.000
	SimSpread_{bin}	0.835	0.963	0.997	0.152	0.664	1.000	0.154	0.667	1.000
	SimSpread_{sim}	0.841	0.972	1.000	0.173	0.705	1.000	0.156	0.667	1.000
GPCR	NN	0.872	0.984	1.000	0.325	0.656	1.000	0.235	0.667	1.000
	SDTNBI	0.895	0.979	1.000	0.378	0.703	1.000	0.333	0.667	1.000
	SimSpread_{bin}	0.887	0.973	1.000	0.302	0.640	1.000	0.250	0.667	1.000
	SimSpread_{sim}	0.875	0.979	1.000	0.313	0.699	1.000	0.254	0.667	1.000
Enzyme	NN	0.898	0.998	1.000	0.210	0.707	1.000	0.105	0.667	1.000
	SDTNBI	0.913	0.998	1.000	0.247	0.706	1.000	0.143	0.667	1.000
	SimSpread_{bin}	0.931	0.995	1.000	0.130	0.728	1.000	0.121	0.704	1.000
	SimSpread_{sim}	0.899	0.989	0.998	0.144	0.816	1.000	0.116	0.800	1.000
Global	NN	0.889	0.992	1.000	0.211	0.577	0.894	0.125	0.500	0.889
	SDTNBI	0.911	0.984	1.000	0.171	0.499	0.866	0.074	0.473	0.857
	SimSpread_{bin}	0.892	0.969	0.995	0.185	0.634	1.000	0.100	0.607	1.000
	SimSpread_{sim}	0.899	0.976	0.998	0.194	0.666	1.000	0.105	0.667	1.000

S4.2 10-times 10-fold cross-validation

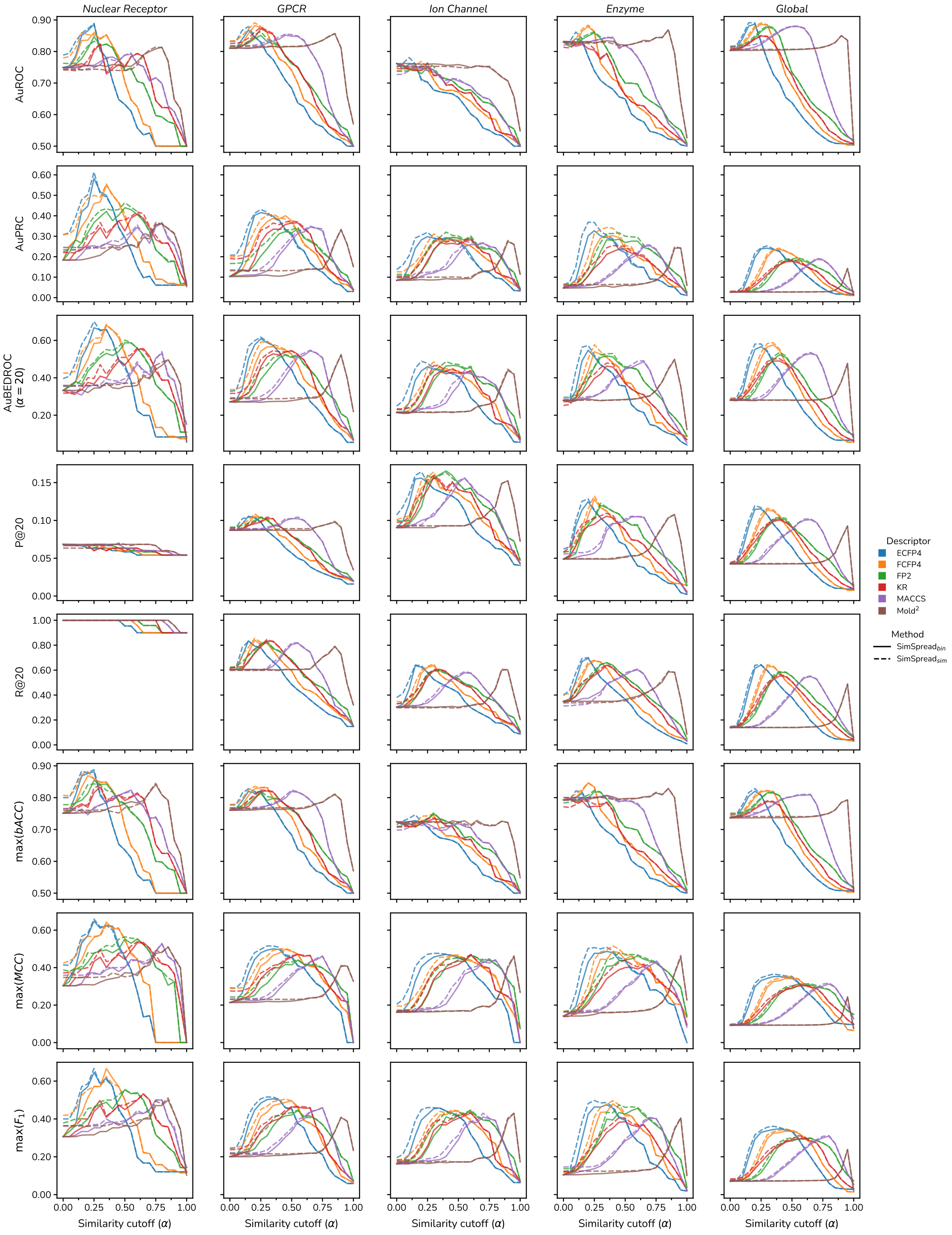

Figure S4: α **parameter optimization over 10-times 10-fold CV.** Median score for metrics obtained for 10-times 10-fold CV over 5 datasets using values between 0 and 1 with a step size of 0.05. A total of six different molecular descriptors were used for the method optimization: ECFP4 (blue), FCFP4 (orange), FP2 (green), KR (red), MACCS keys (purple) and Mold2 (brown). Solid lines correspond to SimSpread$_{bin}$ while dashed lines correspond to SimSpread$_{sim}$

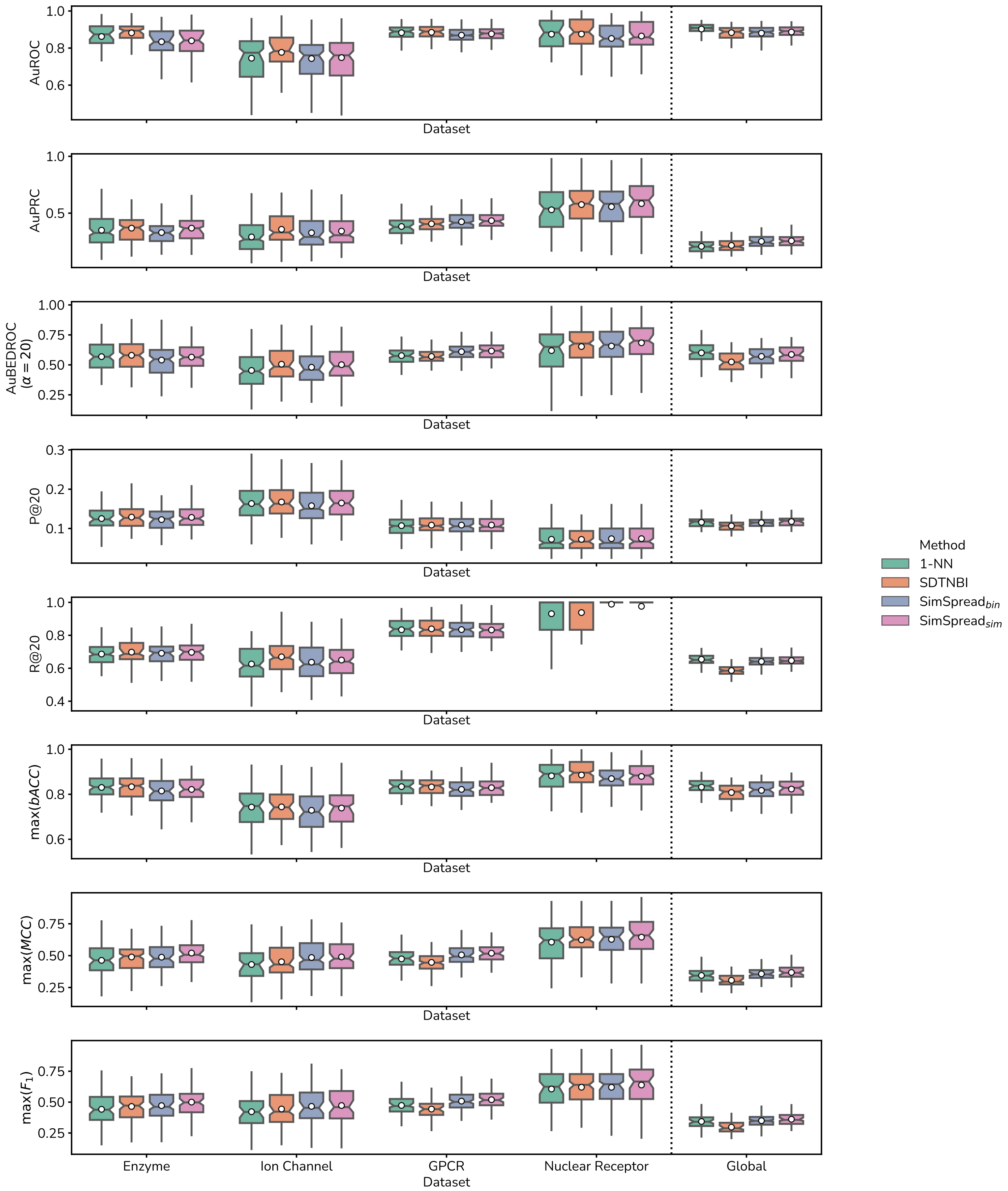

Figure S5: **Predictive performance comparison between the studied methods in 10-times 10-fold CV.** Boxplots for AuROC, AuPRC, AuBEDROC, P@20, R@20, max(MCC), max(F_1) and max(bACC) obtained in the 10-times 10-fold CV over the five validation datasets for 1-NN, NBI, SDTNBI, SimSpread$_{bin}$ and SimSpread$_{sim}$ using ECFP4 molecular descriptor and Tanimoto coefficient as similarity measure. For SimSpread variants, the similarity cutoff is equal to the optimal cutoff that maximizes the performance of the method for that metric. Boxplot notch represents the bootstrapped 95% CI around the median,whiskers correspond to 1.5x IQR and white dot represents the mean.

Table S7: **Overall average performance for the studied methods in 10-times 10-fold CV.** Mean ± SD for AuROC and AuPRC observed in 10-times 10-fold CV over the five validation datasets using the ECFP4 molecular descriptor and Tanimoto coefficient as chemical similarity measure. For SimSpread variants, the similarity cutoff is equal to the optimal cutoff that maximizes the mean performance of the method for that metric.

Dataset	Method	AuROC	AuPRC
NR	NN	0.875 ± 0.090	0.530 ± 0.198
	SDTNBI	0.876 ± 0.092	0.575 ± 0.181
	SimSpread_{bin}	0.852 ± 0.082	0.556 ± 0.188
	SimSpread_{sim}	0.865 ± 0.084	0.584 ± 0.201
IC	NN	0.745 ± 0.120	0.291 ± 0.135
	SDTNBI	0.777 ± 0.108	0.358 ± 0.139
	SimSpread_{bin}	0.743 ± 0.109	0.327 ± 0.135
	SimSpread_{sim}	0.748 ± 0.115	0.342 ± 0.132
GPCR	NN	0.883 ± 0.040	0.382 ± 0.079
	SDTNBI	0.885 ± 0.037	0.406 ± 0.073
	SimSpread_{bin}	0.870 ± 0.037	0.424 ± 0.078
	SimSpread_{sim}	0.877 ± 0.038	0.434 ± 0.080
Enzyme	NN	0.862 ± 0.066	0.350 ± 0.126
	SDTNBI	0.882 ± 0.051	0.368 ± 0.112
	SimSpread_{bin}	0.834 ± 0.072	0.329 ± 0.103
	SimSpread_{sim}	0.839 ± 0.073	0.368 ± 0.121
Global	NN	0.903 ± 0.031	0.208 ± 0.054
	SDTNBI	0.883 ± 0.034	0.216 ± 0.050
	SimSpread_{bin}	0.881 ± 0.036	0.252 ± 0.048
	SimSpread_{sim}	0.886 ± 0.031	0.256 ± 0.049

Table S8: **Overall median performance for the studied methods in 10-times 10-fold CV.** Q1, median and Q3 for AuROC and AuPRC observed in 10-times 10-fold CV over the five validation datasets using the ECFP4 molecular descriptor and Tanimoto coefficient as chemical similarity measure. For SimSpread variants, the similarity cutoff is equal to the optimal cutoff that maximizes the mean performance of the method for that metric.

		AuROC			AuPRC		
Dataset	Method	Q1	median	Q3	Q1	median	Q3
NR	NN	0.809	0.884	0.948	0.379	0.540	0.685
	SDTNBI	0.824	0.886	0.954	0.453	0.583	0.696
	SimSpread_{bin}	0.808	0.851	0.921	0.428	0.583	0.690
	SimSpread_{sim}	0.818	0.858	0.941	0.467	0.613	0.739
IC	NN	0.645	0.775	0.837	0.184	0.268	0.395
	SDTNBI	0.727	0.783	0.857	0.266	0.330	0.473
	SimSpread_{bin}	0.661	0.761	0.817	0.223	0.290	0.431
	SimSpread_{sim}	0.652	0.761	0.828	0.243	0.305	0.430
GPCR	NN	0.861	0.889	0.911	0.324	0.378	0.435
	SDTNBI	0.863	0.889	0.914	0.358	0.404	0.449
	SimSpread_{bin}	0.845	0.868	0.898	0.371	0.415	0.484
	SimSpread_{sim}	0.853	0.879	0.902	0.389	0.430	0.483
Enzyme	NN	0.827	0.874	0.918	0.242	0.326	0.449
	SDTNBI	0.855	0.894	0.917	0.267	0.373	0.439
	SimSpread_{bin}	0.788	0.833	0.890	0.254	0.327	0.386
	SimSpread_{sim}	0.784	0.841	0.894	0.279	0.367	0.433
Global	NN	0.892	0.909	0.925	0.164	0.205	0.245
	SDTNBI	0.856	0.888	0.909	0.175	0.206	0.254
	SimSpread_{bin}	0.862	0.887	0.909	0.212	0.242	0.291
	SimSpread_{sim}	0.871	0.891	0.912	0.218	0.253	0.289

Table S9: **Early-recognition average performance for the studied methods in 10-times 10-fold CV.** Mean ± SD for AuBEDROC, P@20 and R@20 observed in 10-times 10-fold CV over the five validation datasets using the ECFP4 molecular descriptor and Tanimoto coefficient as chemical similarity measure. For SimSpread variants, the similarity cutoff is equal to the optimal cutoff that maximizes the performance of the method for that metric.

Dataset	Method	AuBEDROC	P@20	R@20
NR	NN	0.619 ± 0.191	0.072 ± 0.031	0.931 ± 0.104
	SDTNBI	0.653 ± 0.166	0.072 ± 0.029	0.938 ± 0.097
	SimSpread_{bin}	0.657 ± 0.166	0.074 ± 0.030	0.989 ± 0.044
	SimSpread_{sim}	0.684 ± 0.177	0.074 ± 0.030	0.976 ± 0.054
IC	NN	0.453 ± 0.147	0.164 ± 0.048	0.626 ± 0.103
	SDTNBI	0.505 ± 0.144	0.167 ± 0.043	0.669 ± 0.096
	SimSpread_{bin}	0.480 ± 0.144	0.158 ± 0.047	0.637 ± 0.105
	SimSpread_{sim}	0.501 ± 0.146	0.165 ± 0.047	0.650 ± 0.103
GPCR	NN	0.577 ± 0.069	0.107 ± 0.025	0.834 ± 0.068
	SDTNBI	0.570 ± 0.061	0.109 ± 0.025	0.839 ± 0.061
	SimSpread_{bin}	0.610 ± 0.070	0.109 ± 0.026	0.835 ± 0.064
	SimSpread_{sim}	0.616 ± 0.070	0.109 ± 0.026	0.833 ± 0.061
Enzyme	NN	0.568 ± 0.122	0.125 ± 0.028	0.686 ± 0.063
	SDTNBI	0.580 ± 0.123	0.129 ± 0.028	0.698 ± 0.070
	SimSpread_{bin}	0.540 ± 0.130	0.123 ± 0.028	0.691 ± 0.066
	SimSpread_{sim}	0.565 ± 0.111	0.128 ± 0.029	0.697 ± 0.065
Global	NN	0.600 ± 0.079	0.116 ± 0.011	0.654 ± 0.033
	SDTNBI	0.524 ± 0.080	0.107 ± 0.012	0.586 ± 0.031
	SimSpread_{bin}	0.571 ± 0.078	0.115 ± 0.012	0.641 ± 0.031
	SimSpread_{sim}	0.587 ± 0.078	0.118 ± 0.012	0.647 ± 0.031

Table S10: **Early-recognition median performance for the studied methods in 10-times 10-fold CV.** Q1, median and Q3 for AuBEDROC, P@20 and R@20 observed in 10-times 10-fold CV over the five validation datasets using the ECFP4 molecular descriptor and Tanimoto coefficient as chemical similarity measure. For SimSpread variants, the similarity cutoff is equal to the optimal cutoff that maximizes the performance of the method for that metric.

		AuBEDROC			P@20			R@20		
Dataset	Method	Q1	median	Q3	Q1	median	Q3	Q1	median	Q3
NR	NN	0.486	0.651	0.754	0.050	0.063	0.100	0.833	1.000	1.000
	SDTNBI	0.561	0.678	0.774	0.050	0.067	0.094	0.833	1.000	1.000
	SimSpread_{bin}	0.567	0.668	0.777	0.050	0.063	0.100	1.000	1.000	1.000
	SimSpread_{sim}	0.590	0.701	0.806	0.050	0.067	0.100	1.000	1.000	1.000
IC	NN	0.342	0.447	0.565	0.133	0.162	0.196	0.550	0.616	0.718
	SDTNBI	0.403	0.485	0.617	0.138	0.163	0.198	0.594	0.664	0.735
	SimSpread_{bin}	0.375	0.460	0.571	0.126	0.150	0.191	0.552	0.624	0.726
	SimSpread_{sim}	0.410	0.489	0.609	0.136	0.164	0.196	0.570	0.641	0.712
GPCR	NN	0.525	0.574	0.620	0.089	0.107	0.123	0.796	0.838	0.888
	SDTNBI	0.534	0.564	0.608	0.095	0.107	0.126	0.797	0.833	0.890
	SimSpread_{bin}	0.565	0.609	0.653	0.093	0.106	0.124	0.793	0.835	0.876
	SimSpread_{sim}	0.563	0.618	0.662	0.093	0.104	0.124	0.788	0.832	0.869
Enzyme	NN	0.477	0.568	0.669	0.108	0.123	0.146	0.637	0.684	0.729
	SDTNBI	0.485	0.580	0.673	0.107	0.127	0.149	0.655	0.687	0.754
	SimSpread_{bin}	0.435	0.548	0.625	0.102	0.125	0.143	0.643	0.695	0.732
	SimSpread_{sim}	0.492	0.563	0.647	0.109	0.125	0.149	0.651	0.701	0.739
Global	NN	0.548	0.602	0.664	0.106	0.118	0.123	0.633	0.651	0.676
	SDTNBI	0.462	0.522	0.595	0.097	0.107	0.115	0.566	0.585	0.607
	SimSpread_{bin}	0.513	0.567	0.631	0.106	0.115	0.122	0.623	0.640	0.663
	SimSpread_{sim}	0.534	0.580	0.645	0.108	0.120	0.125	0.629	0.644	0.667

Table S11: **Binary prediction average performance for the studied methods in 10-times 10-fold CV.** Mean ± SD for max(bACC), max(MCC) and max(F_1) observed in 10-times 10-fold CV over the five validation datasets using the ECFP4 molecular descriptor and Tanimoto coefficient as chemical similarity measure. For SimSpread variants, the similarity cutoff is equal to the optimal cutoff that maximizes the performance of the method for that metric.

Dataset	Method	max(bACC)	max(MCC)	max(F_1)
NR	NN	0.882 ± 0.070	0.606 ± 0.150	0.606 ± 0.151
	SDTNBI	0.886 ± 0.068	0.624 ± 0.135	0.621 ± 0.138
	SimSpread_{bin}	0.870 ± 0.055	0.628 ± 0.151	0.620 ± 0.157
	SimSpread_{sim}	0.879 ± 0.054	0.645 ± 0.157	0.639 ± 0.167
IC	NN	0.743 ± 0.085	0.431 ± 0.134	0.422 ± 0.137
	SDTNBI	0.744 ± 0.080	0.452 ± 0.128	0.444 ± 0.134
	SimSpread_{bin}	0.729 ± 0.091	0.485 ± 0.134	0.466 ± 0.141
	SimSpread_{sim}	0.739 ± 0.085	0.490 ± 0.127	0.472 ± 0.143
GPCR	NN	0.834 ± 0.037	0.474 ± 0.070	0.473 ± 0.071
	SDTNBI	0.833 ± 0.035	0.447 ± 0.064	0.444 ± 0.062
	SimSpread_{bin}	0.822 ± 0.043	0.506 ± 0.071	0.508 ± 0.071
	SimSpread_{sim}	0.829 ± 0.036	0.519 ± 0.069	0.519 ± 0.070
Enzyme	NN	0.830 ± 0.056	0.463 ± 0.116	0.442 ± 0.118
	SDTNBI	0.833 ± 0.054	0.489 ± 0.103	0.465 ± 0.118
	SimSpread_{bin}	0.815 ± 0.062	0.488 ± 0.104	0.472 ± 0.117
	SimSpread_{sim}	0.822 ± 0.052	0.521 ± 0.105	0.500 ± 0.117
Global	NN	0.832 ± 0.038	0.345 ± 0.055	0.343 ± 0.054
	SDTNBI	0.808 ± 0.037	0.307 ± 0.046	0.297 ± 0.048
	SimSpread_{bin}	0.817 ± 0.041	0.357 ± 0.042	0.350 ± 0.046
	SimSpread_{sim}	0.823 ± 0.041	0.368 ± 0.048	0.361 ± 0.047

Table S12: **Binary prediction median performance for the studied methods in 10-times 10-fold CV.** Q1, median and Q3 for max(bACC), max(MCC) and max(F_1) observed in 10-times 10-fold CV over the five validation datasets using the ECFP4 molecular descriptor and Tanimoto coefficient as chemical similarity measure. For SimSpread variants, the similarity cutoff is equal to the optimal cutoff that maximizes the performance of the method for that metric.

		max(bACC)			max(MCC)			max(F_1)		
Dataset	Method	Q1	median	Q3	Q1	median	Q3	Q1	median	Q3
NR	NN	0.834	0.891	0.931	0.479	0.621	0.715	0.496	0.625	0.727
	SDTNBI	0.853	0.896	0.944	0.565	0.626	0.724	0.522	0.640	0.727
	SimSpread_{bin}	0.839	0.868	0.906	0.545	0.648	0.720	0.526	0.645	0.727
	SimSpread_{sim}	0.844	0.884	0.925	0.553	0.659	0.765	0.525	0.667	0.764
IC	NN	0.677	0.748	0.803	0.341	0.433	0.520	0.330	0.420	0.509
	SDTNBI	0.690	0.742	0.799	0.368	0.431	0.563	0.339	0.423	0.558
	SimSpread_{bin}	0.655	0.722	0.791	0.392	0.466	0.598	0.370	0.454	0.578
	SimSpread_{sim}	0.679	0.747	0.796	0.402	0.476	0.590	0.368	0.459	0.590
GPCR	NN	0.805	0.834	0.862	0.429	0.478	0.528	0.424	0.468	0.526
	SDTNBI	0.805	0.839	0.862	0.398	0.445	0.496	0.396	0.443	0.487
	SimSpread_{bin}	0.793	0.820	0.853	0.451	0.493	0.559	0.456	0.502	0.561
	SimSpread_{sim}	0.797	0.828	0.857	0.470	0.516	0.566	0.475	0.516	0.568
Enzyme	NN	0.799	0.832	0.870	0.385	0.464	0.558	0.356	0.439	0.542
	SDTNBI	0.790	0.838	0.871	0.403	0.497	0.549	0.377	0.472	0.546
	SimSpread_{bin}	0.773	0.814	0.859	0.410	0.477	0.567	0.390	0.462	0.560
	SimSpread_{sim}	0.788	0.821	0.865	0.448	0.507	0.582	0.418	0.498	0.567
Global	NN	0.818	0.838	0.859	0.305	0.343	0.380	0.306	0.341	0.377
	SDTNBI	0.779	0.812	0.838	0.274	0.296	0.342	0.263	0.288	0.333
	SimSpread_{bin}	0.791	0.820	0.853	0.325	0.353	0.387	0.317	0.348	0.380
	SimSpread_{sim}	0.798	0.829	0.856	0.334	0.365	0.406	0.324	0.356	0.396

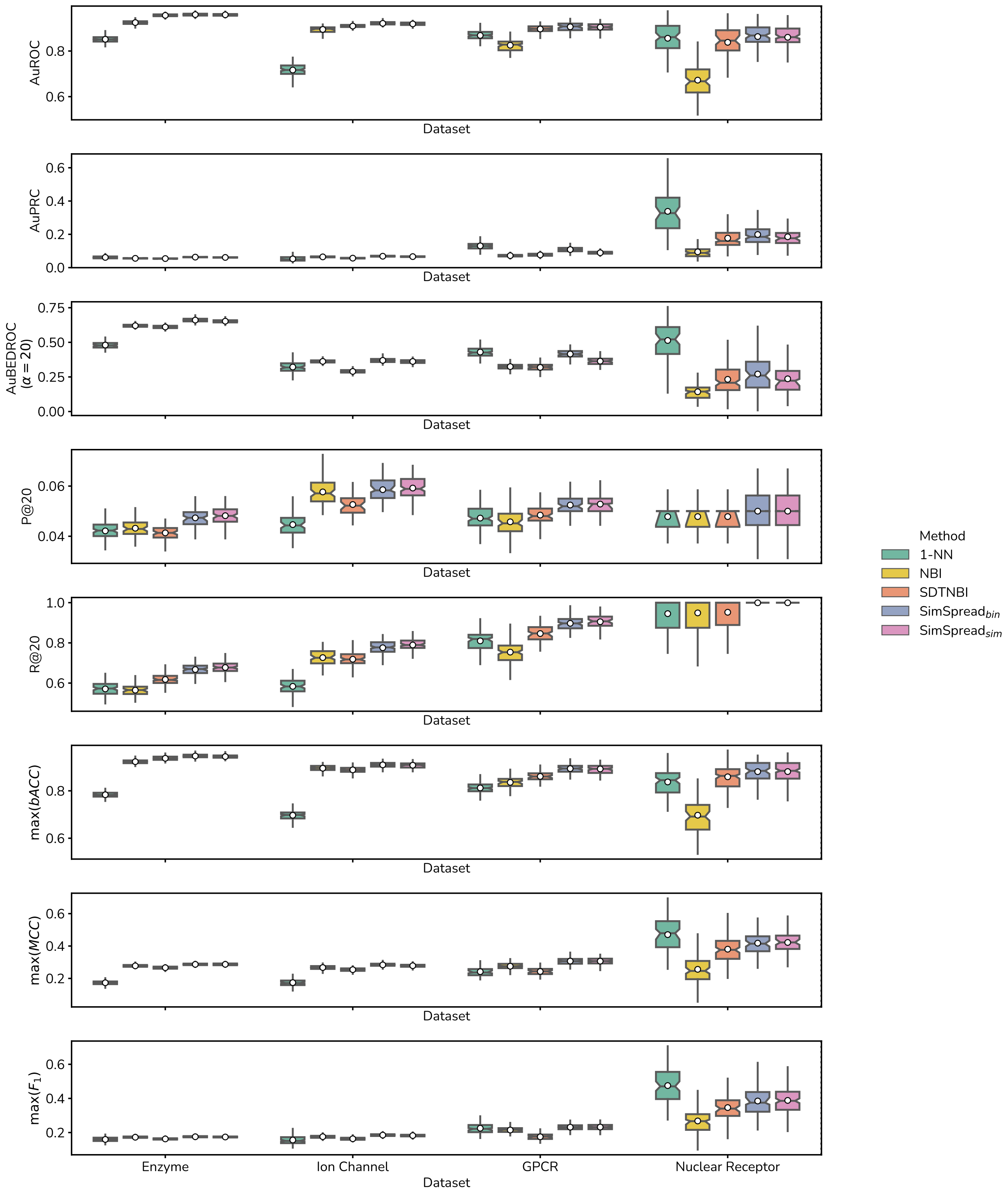

Figure S6: **Predictive performance comparison between the studied methods in 10-times 10-fold CV using DTI holdout.** Boxplots for AuROC, AuPRC, AuBEDROC, P@20, R@20, max(MCC), max(F_1) and max(bACC) obtained in the 10-times 10-fold CV using DTI holdout over the five validation datasets for 1-NN, NBI, SDTNBI, SimSpread$_{bin}$ and SimSpread$_{sim}$ using ECFP4 molecular descriptor and Tanimoto coefficient as similarity measure. For SimSpread variants, the similarity cutoff is equal to the optimal cutoff that maximizes the performance of the method for that metric. Boxplot notch represents the bootstrapped 95% CI around the median,whiskers correspond to 1.5x IQR and white dot represents the mean.

S4.3 Time-split cross-validation

Table S13: **Overall average performance for the studied methods in time-split CV.** Mean ± SD for AuROC and AuPRC observed in time-split CV using the ECFP4 molecular descriptor and Tanimoto coefficient as chemical similarity measure. For SimSpread variants, the similarity cutoff α is equal to 0.2.

Method	AuROC	AuPRC
1-NN	0.807 ± 0.261	0.303 ± 0.352
SDTNBI	0.765 ± 0.266	0.252 ± 0.335
SimSpread$_{bin}$	0.774 ± 0.264	0.279 ± 0.349
SimSpread$_{sim}$	0.778 ± 0.265	0.310 ± 0.367

Table S14: **Overall median performance for the studied methods in time-split CV.** Q1, median and Q3 for AuROC and AuPRC observed in time-split CV using the ECFP4 molecular descriptor and Tanimoto coefficient as chemical similarity measure. For SimSpread variants, the similarity cutoff α is equal to 0.2.

	AuROC			AuPRC		
Method	Q1	median	Q3	Q1	median	Q3
1-NN	0.699	0.942	0.997	0.014	0.137	0.500
SDTNBI	0.623	0.873	0.990	0.009	0.067	0.417
SimSpread$_{bin}$	0.500	0.912	0.994	0.009	0.090	0.500
SimSpread$_{sim}$	0.500	0.932	0.995	0.01	0.111	0.539

Table S15: **Early-recognition average performance for the studied methods in time-split CV.** Mean ± SD for AuBEDROC, P@20 and R@20 observed in time-split CV using the ECFP4 molecular descriptor and Tanimoto coefficient as chemical similarity measure. For SimSpread variants, the similarity cutoff α is equal to 0.2.

Method	AuBEDROC	P@20	R@20
1-NN	0.496 ± 0.409	0.065 ± 0.092	0.526 ± 0.464
SDTNBI	0.405 ± 0.399	0.055 ± 0.079	0.442 ± 0.457
SimSpread$_{bin}$	0.451 ± 0.402	0.061 ± 0.085	0.497 ± 0.464
SimSpread$_{sim}$	0.476 ± 0.410	0.063 ± 0.086	0.517 ± 0.463

Table S16: **Early-recognition median performance for the studied methods in time-split CV.** Q1, median and Q3 for AuBEDROC, P@20 and R@20 observed in time-split CV using the ECFP4 molecular descriptor and Tanimoto coefficient as chemical similarity measure. For SimSpread variants, the similarity cutoff α is equal to 0.2.

	AuBEDROC			P@20			R@20		
Method	Q1	median	Q3	Q1	median	Q3	Q1	median	Q3
1-NN	0.016	0.527	0.951	0.000	0.050	0.100	0.000	0.667	1.000
SDTNBI	0.002	0.305	0.840	0.000	0.050	0.050	0.000	0.333	1.000
SimSpread$_{bin}$	0.004	0.434	0.885	0.000	0.050	0.100	0.000	0.500	1.000
SimSpread$_{sim}$	0.006	0.477	0.909	0.000	0.050	0.100	0.000	0.500	1.000

Table S17: **Binary prediction average performance for the studied methods in time-split CV.** Mean ± SD for max(bACC), max(MCC) and max(F_1) observed in time-split CV using the ECFP4 molecular descriptor and Tanimoto coefficient as chemical similarity measure. For SimSpread variants, the similarity cutoff α is equal to 0.2.

Method	max(bACC)	max(MCC)	max(F_1)
1-NN	0.885 ± 0.139	0.418 ± 0.341	0.370 ± 0.361
SDTNBI	0.861 ± 0.145	0.356 ± 0.329	0.309 ± 0.343
SimSpread$_{bin}$	0.829 ± 0.201	0.376 ± 0.350	0.339 ± 0.357
SimSpread$_{sim}$	0.831 ± 0.201	0.405 ± 0.365	0.372 ± 0.373

Table S18: **Binary prediction median performance for the studied methods in time-split CV.** Q1, median and Q3 for max(bACC), max(MCC) and max(F_1) observed in time-split CV using the ECFP4 molecular descriptor and Tanimoto coefficient as chemical similarity measure. For SimSpread variants, the similarity cutoff α is equal to 0.2.

	max(bACC)			max(MCC)			max(F_1)		
Method	Q1	median	Q3	Q1	median	Q3	Q1	median	Q3
1-NN	0.811	0.949	0.997	0.099	0.347	0.706	0.030	0.250	0.667
SDTNBI	0.755	0.905	0.994	0.073	0.242	0.576	0.020	0.143	0.500
SimSpread$_{bin}$	0.643	0.942	0.996	0.048	0.273	0.705	0.017	0.190	0.667
SimSpread$_{sim}$	0.646	0.947	0.997	0.049	0.313	0.706	0.019	0.222	0.667

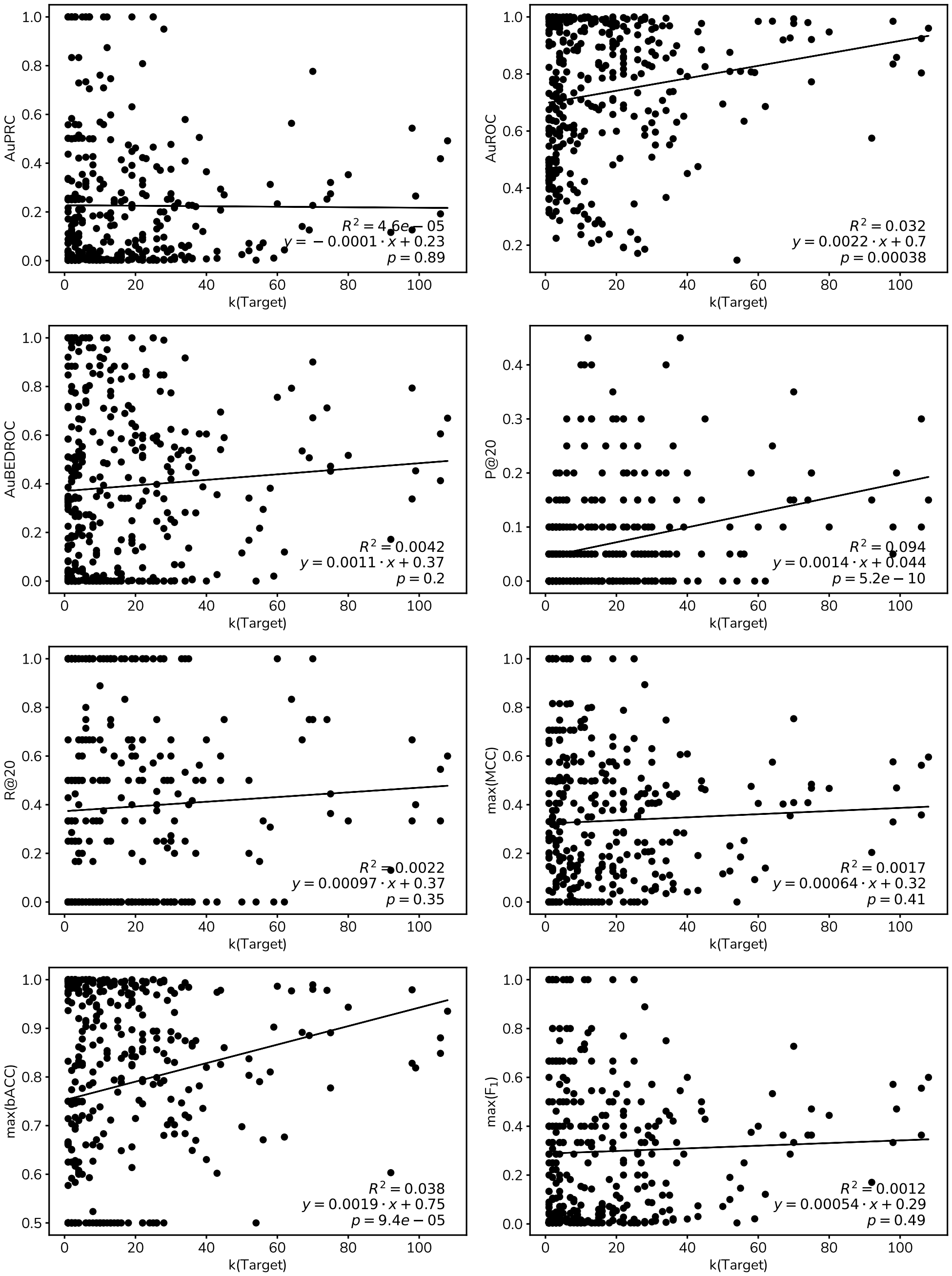

Figure S7: **Relationship between target degree and predictive performance.** Per target performance for SimSpread$_{sim}$ over the ChEMBL CC&D time-split validation using the ECFP4 molecular descriptor. R^2 and p corresponds to the coefficient of determination and p-value for a hypothesis test that the slope is nonzero (Wald Test with t-distribution) obtained from the linear least-squares regression between target degree and predictive performance over the 8 evaluation metrics used in the study.

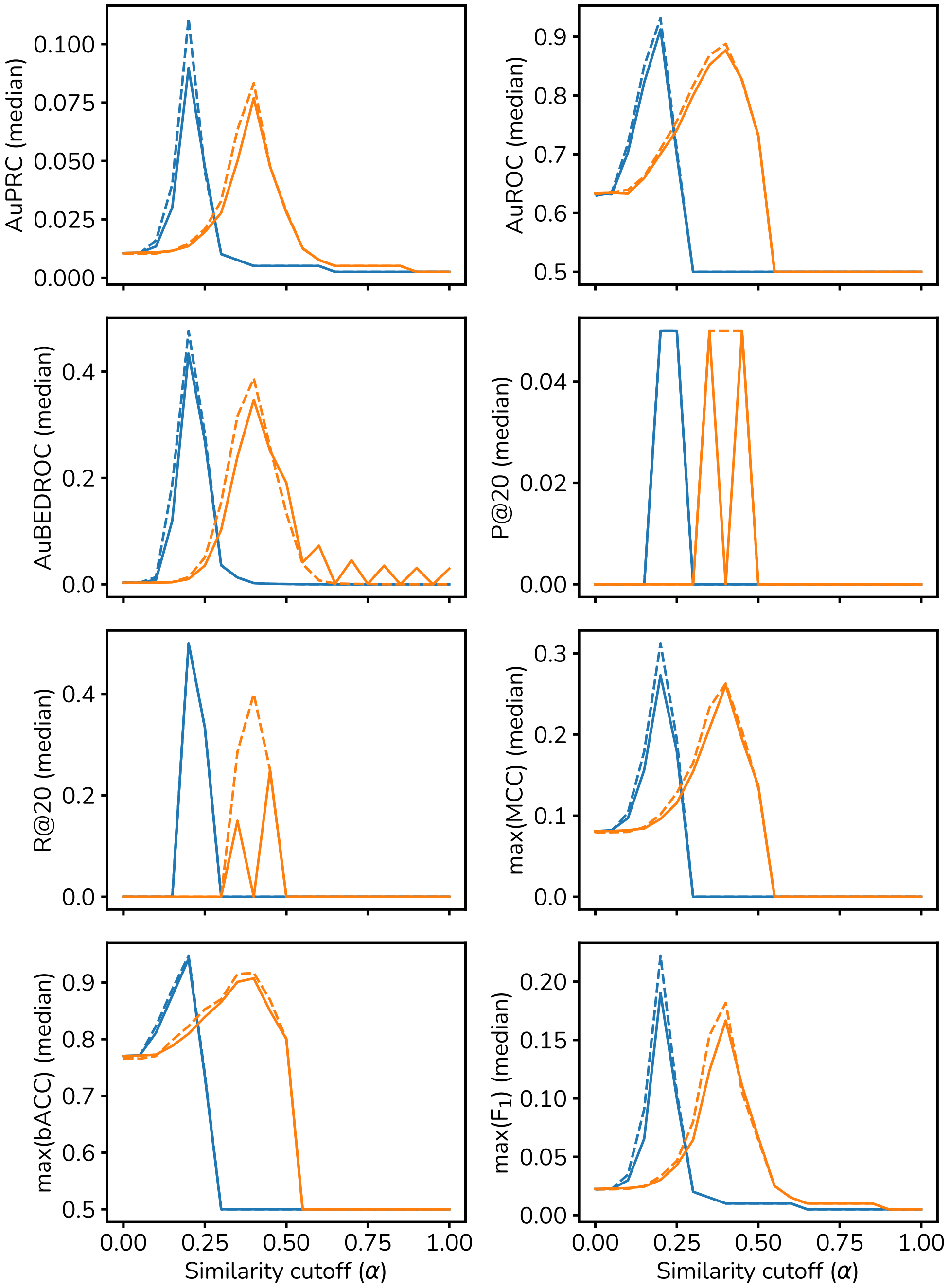

Figure S8: **Relationship between similarity measure and predictive performance for time-split validation.** SimSpread performance obtained for the ChEMBL CC&D time-split validation using the ECFP4 molecular descriptor using 2 similarity measures: Tanimoto coefficient (in blue) and Tversky index with $\alpha = 0.0$ and $\beta = 1.0$ (in orange). Solid line corresponds to SimSpread$_{bin}$ and dashed lines correspond to SimSpread$_{sim}$.

S5 Computation time

Table S19: **SimSpread$_{sim}$ computation time for 10-times 10-fold cross-validation**

Dataset	Mean time per 10-fold CV (seconds)	Mean time per fold (seconds)
Nuclear Receptor	7.01	0.701
Ion Channel	9.727	0.973
GPCR	8.671	0.867
Enzyme	21.838	2.184
Global	140.137	14.137

Note: Computation times where detemined using the `@elapsed` macro in Julia Language version 1.7.3. Benchmark was run on the following hardware using GPU acceleration through `CUDA.jl` package: Intel(R) Xeon(R) CPU E5-2697 v4 @ 2.30GHz, 536 GB RAM & NVIDIA GeForce GTX 1080-Ti 12Gb VRAM.

S6 Reimplementation of benchmark methods

Table S20: Reimplementation of SDTNBI using the Global dataset with Klethora-Roth descriptor in LOO cross-validation

Implementation	AuROC	P@20	R@20
In-house	0.911 ± 0.150	0.097 ± 0.165	0.500 ± 0.351
Wu, et al (2016)	0.910 ± 0.150	0.099 ± 0.167	0.493 ± 0.438

Note: Values correspond to mean ± SD over the predictive performance of each fold.

Table S21: Reimplementation of 1-NN using the ChEMBL24→28 dataset with ECFP4 descriptor under time-split cross-validation

Implementation	AuROC	AuPRC
Julia (network-based)	0.81056	0.13229
C++	0.81056	0.13230

Note: Julia (network-based): Our network-based k-NN algorithm implemented in-house in Julia, which was employed throughout this study. C++: Reference k-NN algorithm implemented in-house in C++ based on the works of Gfeller et al. (2013) [3].

S7 Examples

Table S22: Target prediction for Nintedanib and Pirfenidone over ChEMBL28 CC&D network

Compound	Score	Rank	Target ChEMBL ID	Target Name
Nintedanib	0.006781	1	CHEMBL279	Vascular endothelial growth factor receptor 2
	0.005965	2	CHEMBL3650	Fibroblast growth factor receptor 1
	0.005891	3	CHEMBL1955	Vascular endothelial growth factor receptor 3
	0.005730	4	CHEMBL2007	Platelet-derived growth factor receptor alpha
	0.005473	5	CHEMBL1913	Platelet-derived growth factor receptor beta
	0.005406	6	CHEMBL2742	Fibroblast growth factor receptor 3
	0.005404	7	CHEMBL3973	Fibroblast growth factor receptor 4
	0.005393	8	CHEMBL4142	Fibroblast growth factor receptor 2
	0.005345	9	CHEMBL1868	Vascular endothelial growth factor receptor 1
	0.003402	10	CHEMBL240	Potassium voltage-gated channel subfamily H member 2
	0.003377	11	CHEMBL238	Sodium-dependent dopamine transporter
	0.002833	12	CHEMBL1974	Receptor-type tyrosine-protein kinase FLT3
	0.002408	13	CHEMBL258	Tyrosine-protein kinase Lck
	0.002180	14	CHEMBL267	Proto-oncogene tyrosine-protein kinase Src
	0.002103	15	CHEMBL3905	Tyrosine-protein kinase Lyn
Pirfenidone	0.027070	1	CHEMBL4523354	Nicotinate phosphoribosyltransferase
	0.007884	2	CHEMBL4523441	Deoxyhypusine hydroxylase
	0.006112	3	CHEMBL4596	C-C chemokine receptor type 8
	0.004195	4	CHEMBL214	5-hydroxytryptamine receptor 1A
	0.003968	5	CHEMBL238	Sodium-dependent dopamine transporter
	0.003903	6	CHEMBL228	Sodium-dependent serotonin transporter
	0.003880	7	CHEMBL4685	Indoleamine 2,3-dioxygenase 1
	0.003740	8	CHEMBL3242	Carbonic anhydrase 12
	0.003641	9	CHEMBL3155	5-hydroxytryptamine receptor 7
	0.003485	10	CHEMBL2009	Glutamate receptor 1
	0.003128	11	CHEMBL205	Carbonic anhydrase 2
	0.002962	12	CHEMBL222	Sodium-dependent noradrenaline transporter
	0.002913	13	CHEMBL220	Acetylcholinesterase
	0.002761	14	CHEMBL4295747	Serine hydroxymethyltransferase, mitochondrial
	0.002761	14	CHEMBL4523129	Synaptojanin-2
	0.002761	14	CHEMBL4523136	Synaptojanin-1

Note: Prediction for Nintedanib and Pirfenidone where obtained using SimSpread$_{sim}$ with the ECFP4 molecular descriptor and a similarity cutoff α equal to 0.2. Query network corresponds to ChEMBL28 CC&D dataset from which the time-split datasets where constucted.

References

1. Mold2 — FDA. Available online: https://www.fda.gov/science-research/bioinformatics-tools/mold2 (accessed on 27 July 2022)

2. Hong, et al (2008). Mold2, Molecular Descriptors from 2D Structures for Chemoinformatics and Toxicoinformatics. Journal of Chemical Information and Modeling, 48(7), 1337–1344. https://doi.org/10.1021/ci800038f

3. Gfeller et al (2013). Shaping the interaction landscape of bioactive molecules. Bioinformatics, 29(23), 3073-3079. https//doi.org/10.1093/bioinformatics/btt540
